# In-Situ Synthesis of TiO_2_@GO Nanosheets for Polymers Degradation in a Natural Environment

**DOI:** 10.3390/polym13132158

**Published:** 2021-06-30

**Authors:** Yueqin Shi, Zhanyang Yu, Zhengjun Li, Xiaodong Zhao, Yongjun Yuan

**Affiliations:** College of Materials and Environmental Engineering, Hangzhou Dianzi University, Xiasha Higher Education Zone, Hangzhou 310018, China; 18205131@hdu.edu.cn (Z.Y.); lzj18205118@hdu.edu.cn (Z.L.); xdzhao@hdu.edu.cn (X.Z.); yjyuan@hdu.edu.cn (Y.Y.)

**Keywords:** polymers degradation, natural environment, photocatalyst, compounds

## Abstract

Plastic photodegradation naturally takes 300–500 years, and their chemical degradation typically needs additional energy or causes secondary pollution. The main components of global plastic are polymers. Hence, new technologies are urgently required for the effective decomposition of the polymers in natural environments, which lays the foundation for this study on future plastic degradation. This study synthesizes the in-situ growth of TiO_2_ at graphene oxide (GO) matrix to form the TiO_2_@GO photocatalyst, and studies its application in conjugated polymers’ photodegradation. The photodegradation process could be probed by UV-vis absorption originating from the conjugated backbone of polymers. We have found that the complete decomposition of various polymers in a natural environment by employing the photocatalyst TiO_2_@GO within 12 days. It is obvious that the TiO_2_@GO shows a higher photocatalyst activity than the TiO_2_, due to the higher crystallinity morphology and smaller size of TiO_2_, and the faster transmission of photogenerated electrons from TiO_2_ to GO. The stronger fluorescence (FL) intensity of TiO_2_@GO compared to TiO_2_ at the terephthalic acid aqueous solution indicates that more hydroxyl radicals (•OH) are produced for TiO_2_@GO. This further confirms that the GO could effectively decrease the generation of recombination centers, enhance the separation efficiency of photoinduced electrons and holes, and increase the photocatalytic activity of TiO_2_@GO. This work establishes the underlying basic mechanism of polymers photodegradation, which might open new avenues for simultaneously addressing the white pollution crisis in a natural environment.

## 1. Introduction

The decomposition process of plastics required approximately 300–500 years, due to their strong chemical stability, which led to serious global environmental problems. Especially, the ingredients of plastics could invade the animals, crops, and even marine creatures, which might eventually enter the human body through the food chain transmission, and finally menace human health [1,2,3,4]. While the main components of global plastic were the polymers, hence, it was urgently required new technologies for the effective degradation of the polymers in a natural environment, which laid the foundation for the study of future plastic degradation.

The thermocatalytic decomposition, alkali solution, and photocatalytic oxidation ways had been reported to decompose the polymers in addition to the landfill and incineration [5]. However, thermocatalytic degradation usually demanded high temperatures (>400 °C) and precious metal complex catalysts to narrow the product distribution, which limited their practical applications [6]. The polymers could be initially hydrolyzed into organic products at alkali solution, but the following photooxidation process would result in a variety of small carbonaceous molecules. Besides, the residual solution with strong alkalinity inevitably had detrimental effects on the natural environment [7]. It was highly imperative to find innovative solutions for the polymers’ effective degradation via more environmentally-friendly ways in a natural environment.

Photocatalytic oxidation was currently the most promising and environmentally-friendly method for the polymers’ photodegradation [5,6,7,8,9]. The photocatalytic oxidation method was the catalyst that absorbed energy to undergo an electron transition, generating free radicals or empty orbitals under light illumination, which could easily accept electrons and demonstrated strong oxidizing properties. The optimized catalyst for polymers degradation endowed with the characteristics, non-toxic, low price, high chemical stability, water-insoluble (benefit for recycling and reusing) and high photocatalytic activity, etc. The commonly used photocatalysts were transition metal oxides [10]. The transition metal oxides had the characteristics of heat resistance, non-toxicity, stability, semiconductor, and even the d orbital electron layer of its metal cations was prone to lose or gain electrons, thus displaying strong redox ability [10,11,12,13,14,15].

TiO_2_ had been widely used in organic molecule degradation, such as methylene blue, owing to its innocuousness, stability, and high photocatalytic efficiency [11]. However, the low absorption ratio of solar light and weak separation efficiency of photocarries were the important factors in restricting the catalytic activity of TiO_2_ applied in polymers degradation [10]. The compounds of TiO_2_ and carbonaceous materials with different shapes and structures were reported to improve the photocatalytic activity of TiO_2_ [12,13]. Because the energy band edge of TiO_2_ could be enhanced via changing the surface microstructure, doping, or introducing carbide structures in different local environments to meet all the redox potentials involved in the photooxidation process [10,11,12]. Meanwhile, the carbon materials could reduce the recombination efficiency of carriers generated on TiO_2_ by promoting the transmission of photogenerated electrons [12,13,14,15]. The carbon materials not only improved the adsorption capacity of target pollutants on the surface of TiO_2_, but also decreased the generation of recombination centers of TiO_2_ by promoting the transmission of photogenerated electrons [8,9].

Graphene oxide (GO) was a derivative of graphene [13]. Due to the introduction of a large number of oxygen-active functional groups (hydroxyl, epoxy, carbonyl, carboxyl, etc.) on the graphene sheet, the relative inert surface of graphene became extremely active. It was easier to achieve the self-assembled TiO_2_ at the surface of GO through reacting with the active functional groups. The compound of TiO_2_ and GO could be assumed as an ideal photocatalyst for polymers degradation by combining the high electron-transport property of GO with the highly photocatalytic activity of TiO_2_.

Herein, we provided an in-situ method to synthesize TiO_2_ using the GO matrix to achieve the TiO_2_@GO photocatalyst and applied the TiO_2_@GO as photocatalyst for the photodegradation of polymers with the illumination of natural light. The X-ray Diffractomer (XRD) and Scanning Electron Microscope (SEM) analysis were carried out to identify the crystalline structure of the photocatalyst. The photodegradation process of polymers was detected by UV-vis absorption. We had found the complete decomposition of various polymers within 12 h in a natural environment by employing the photocatalyst TiO_2_@GO. The fluorescence (FL) measurement was used to further study the effect of GO on the separation and recombination of photogenerated exciton of TiO_2_ through detecting the production of •OH from polymers at the terephthalic acid aqueous of catalyst. This work established the underlying basic mechanism of polymers photodegradation, which might open new avenues for simultaneously addressing the white pollution crisis in a natural environment.

## 2. Experimental Procedure

### 2.1. Synthesis of All Samples

#### 2.1.1. The Polymers’ Synthesis

All the chemicals here were analytical-grade and used without further treatments (M.K Chemical Industry, Hangzhou, China). The EM Science Silica Gel 60 F254 glass plates were used to perform the thin layer chromatography (TLC) (Sigma-Aldrich^®^, Shanghai, China). And the 60 Å silica gel (37–75 µm) was further applied to perform the Flash chromatography. The polymers dialysis was processed by using the dialysis membranes with the MWCO of 3500–5000 Da, which was bought from Spectrum^®^ Laboratories Inc (Shanghai, China). ^1^H NMR spectra were recorded at either 400 MHz or 500 MHz in D_2_O or CD_3_OD (Varian spectrometer, Walnut Creek, CA, USA). All the monomers synthesis was displayed at the Supplementary Materials. The GPC of all polymers was measured on the Alliance HPLC (Waters, Milford, MA, USA) with PEG standard using pure water as solvent under the condition of column temperature at 40 °C and a flow rate of 1.0 mL/min at Shanghai Institute of Organic Chemistry.

#### 2.1.2. Yamamoto Polymerizations of PBTz-SO_3_Na (P1) and PBTz-TMAI (P2) 

Under the argon atmosphere, an equimolecular amount of dibromo-monomer (1 mol) was mixed to the 1,5-cyclooctadiene (1 mol), 2,2-bipyridine (1 mol) and bis(1,5-cyclooctadiene)nickel(0) (1 mol) solution at 60 °C. The mixture was stirred and reacted at 65 °C for two days. After cooling to room temperature, the product was poured into chloroform and washed with chloroform, acetone, and hexane by Soxhlet extraction, and followed by dissolution in Millipore water. Finally, the solution was further purified in a dialysis tube (MWCO = 3500–5000 Da) through soaking in Millipore water for five days. The water was changed every 12 h. After freeze-drying, the corresponding polymers were obtained as red solids [16,17].

#### 2.1.3. Suzuki Coupling Polymerization of PBTBTz-SO_3_Na (P3) 

Na_2_CO_3_ (5 mol), Pd(PPh_3_)_4_ (2 mol%), dibromide (1 mol), and diboronic ester (1 mol) were added in the microwave tube with a stir bar at the glove box (Mikrouna, Shanghai, China). The tube was sealed and taken out of the glove box. A mixture of DMF and H_2_O (4/1 vol/vol) was degassed by argon for 5 min, then transferred into the above tube. The solution was placed into an oil bath, and heated to 80 °C, at which temperature the mixture turned dark red in ~20 min. The reaction was conducted at ~80 °C for 24 h, and poured into acetone. Red precipitates were collected by filtration and washed with acetone and MeOH. The red products were dissolved in Millipore H_2_O and then transferred into a dialysis tube (MWCO: 3500–5000). The tube was placed into the Millipore H_2_O for dialysis in a week, and the Millipore H_2_O was changed every 12 h. After freeze-drying, the P3 was achieved as a dark red solid [16,17].

**Scheme 1:**
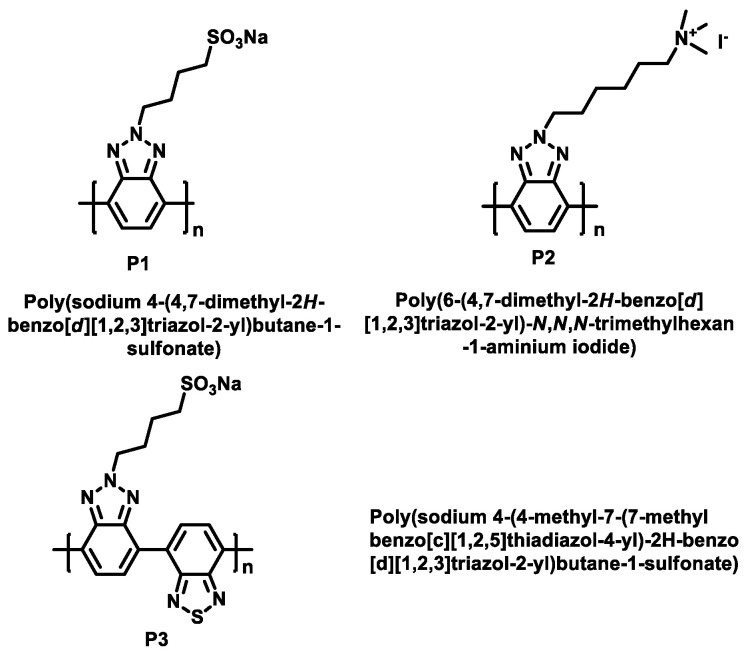
The chemical structure and name of water-soluble conjugated polymers.

The chemical structure and name of water-soluble conjugated polymers were demonstrated in Scheme 1 (P1: *M*_n_ = 172 K, Đ = 3.7. P2: *M*_n_ = 157.5 K, Đ = 1.5. P3: *M*_n_ = 94 K, Đ = 2.8.). The average *M*_n_ and dispersity were obtained by GPC in water that was calibrated versus poly(ethylene glycol) standards, seen the Supplementary Materials, Figures S1 and S2 [16,17].

The synthesis of TiO_2_: 0.72 g Potassium hydroxide was dissolved in 100 mL deionized water. The solution was added 1.4 mL TiCl_4_ in drops and stirred for 3 h. Then keep the reaction at 60 °C for 24 h, and the product (TiO_2_ photocatalyst) was finally obtained by being washed with deionized water and dried at 60 °C.

In-situ growth of TiO_2_ using the GO matrix: 0.1 g GO was suspended in 100 mL deionized water. Then 0.72 g potassium hydroxide was added, and the solution was stirred for 10 min. The solution was added 1.4 mL TiCl_4_ in drops and stirred for 3 h. Then keep the reaction at 60 °C for 24 h, and the product (TiO_2_@GO photocatalyst) was finally obtained by being washed with deionized water and dried at 60 °C.

### 2.2. Characterizations

The crystal structures of all samples were processed by using the XRD (TD-3500; Tongda Instrument Co., Ltd., Dandong, China). The morphology of as-prepared samples was obtained using an SEM (S-4800; Hitachi, Tokyo, Japan), which was equipped with an energy-dispersive X-spectrometer (EDS). The X-ray photoelectron spectroscopy (XPS) of all samples was recorded in a Thermo Fisher ESCALAB 250 xi (Waltham, MA, USA) using AlK^ɑ^ radiation (1486.6 eV). Binding energies were measured with a precision of ±0.05 eV and calculated with respect to C (1s) at 284.8 eV. The decomposition of organic materials was detected by using a UV-vis spectrophotometer (UV-3600; Shimadzu, Tokyo, Japan). The fluorescence (FL) emission spectra of TiO_2_ and TiO_2_@GO were measured on an FL spectrophotometer (RF-530TPC, Shimadzu, Tokyo, Japan).

### 2.3. Photocatalytic Experiments of Methylene Blue

The degradation rate of methylene blue with the presence of the photocatalyst was processed to study the photocatalytic properties of catalysts. The solution of methylene blue and photocatalyst was placed in a dark environment for 120 min to reach the adsorption equilibrium before photodegradation, and then the residual concentration of methylene blue was measured by the UV-vis spectrometry. The lamp was placed 8 cm above the liquid surface, which was the 250 W high-pressure mercury lamp with a 420 nm cutoff filter as the visible light source. A photocatalyst of 0.01 g was added into the methylene blue aqueous solution (100 mL, 1 × 10^−^^5^ M). The mixed solution was stirred, and after every 20 min, 3 mL solution was extracted to test the residual concentration of methylene blue. The concentration of polymer (C) was proportional to the absorption peak of polymer, which could be evaluated by measuring the maximum absorbance of methylene blue in the UV-vis spectrometry.

### 2.4. Photocatalytic Degradation of Polymers

Polymers’ photodecomposition was processed under the illumination of natural light outside at about 0–5 °C [15]. Then, 3 Mg Photocatalyst was added into 20 mL polymers aqueous solutions. The concentration of polymers was 20 mg/L. The solution of polymer and photocatalyst was also placed in a dark environment for 120 min to reach the adsorption equilibrium before photocatalysis experiments to preclude the adsorption of photocatalyst, and the remained concentration of polymer was regarded as the initial value. Photocatalyst was added into the polymer aqueous solution, which was placed outside with the illumination of natural light. After about every 2 days, the solution was measured to test the residual concentration of polymer. The concentration of polymer (C) was proportional to the absorption peak of polymer, which could be evaluated by measuring the maximum absorbance of polymer in UV-vis spectrometry. The stability of catalyst was also studied by the recycle experiments of polymer’s degradation with the reusable catalyst three times.

### 2.5. Hydroxyl Radical (•OH) Measurement

The photocatalyst’s activity was processed through exploring the •OH generating at photoilluminated water/photocatalyst interface by FL measurement using the terephthalic acid as the detecting molecule (RF-530TPC, Shimadzu, Tokyo, Japan). A strong fluorescent product, 2-hydroxyterephthalic acid, would be obtained when the •OH reacted with terephthalic acid. Here, 0.1 g catalyst was mixed into the 100 mL terephthalic acid aqueous solution (3 × 10^−^^3^ M) with the NaOH concentration of 1 × 10^−^^2^ M. The solution was stirred to produce •OH, which could be evaluated by measuring the FL intensity emitted from 2-hydroxyterephthalic acid at about 420 nm excited by 310 nm light.

## 3. Results and Discussion

**Scheme 2:**
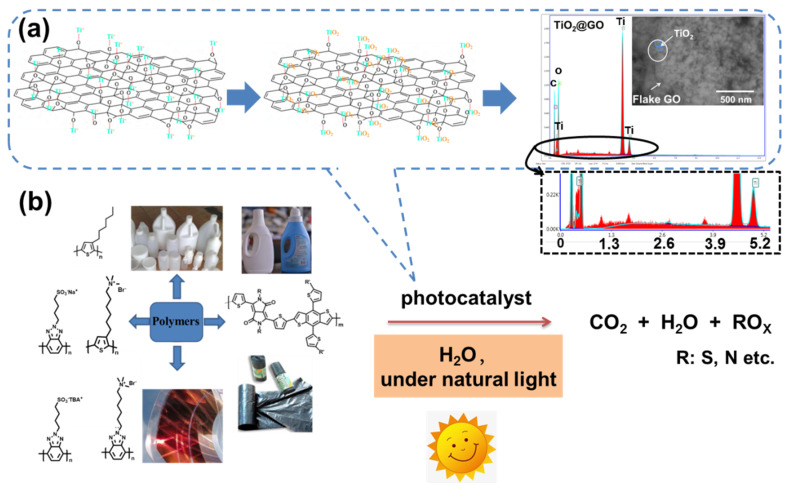
(**a**) The in-situ synthesis of TiO_2_ at GO to form the TiO_2_@GO photocatalyst. (The inset SEM image showed the morphology of TiO_2_@GO and the detailed test area for the EDS spectra of TiO_2_ at GO.) (**b**) Schematic illustration for converting various polymers into CO_2_ with the illumination of natural light through a photooxidative C–C bond cleavage process over the photocatalyst.

Scheme 2 was a proof diagram of the in-situ growth of TiO_2_ using the GO matrix to obtain the TiO_2_@GO photocatalyst. The inset SEM image showed the nanosheets morphology of TiO_2_@GO. The EDS element analysis was performed on the white part of the SEM image, displaying the existence of C, Ti, and O at TiO_2_@GO and the successful synthesis of TiO_2_ nanocrystals self-assembling into a GO matrix. This work established the underlying basic mechanism of various polymers’ photodegradation with the illumination of natural light through a photooxidative C–C bond cleavage process over the photocatalyst, even including the solar devices of lab and the plastics of our life.

**Figure 1:**
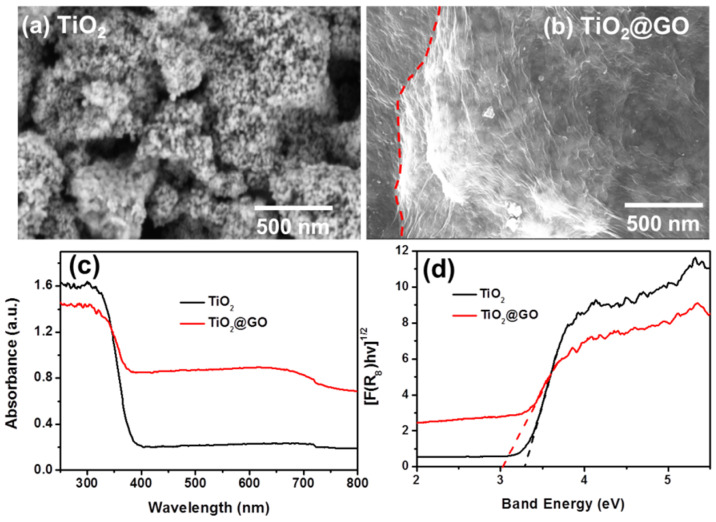
SEM of (**a**) TiO_2_ and (**b**) TiO_2_@GO, (**c**) the UV-vis DRS, and (**d**) the plots of [*F(R_∞_)hv*]^1/2^ vs. photon energy of TiO_2_ and TiO_2_@GO.

The SEM image (Figure 1) was used to study the microstructure of TiO_2_ and TiO_2_@GO photocatalysts. The TiO_2_ sample appeared as porous nanocrystals with a nanoscale diameter. Whereas, it was observed that the TiO_2_ nanocrystals, with about 20–50 nm diameter, were well-assembled into the GO nanosheets matrix for the TiO_2_@GO sample. The nanostructure with interconnected nanosheets of TiO_2_@GO benefitted from facilitating the better intimate charge transfer between TiO_2_ and GO with the illumination of natural light [18]. The band gaps of TiO_2_ and TiO_2_@GO were detected by UV-vis diffuse reflectance spectra in Figure 1 [19]. Compared with pure TiO_2_, TiO_2_@GO showed stronger visible light absorption. To further study the energy band gap of TiO_2_ and TiO_2_@GO, the intercept of the tangents to the plots of [*F(R_∞_)hv*]^1/2^ vs. photon energy (*hv*) was demonstrated. The Kubelka-Munk function *F(R_∞_)* and photon energy (*hv*) could be estimated by the equation as *F(R_∞_)* = (1 − *R_∞_*)^2^/2*R_∞_*, (*R_∞_* = 10*^−A^*, *hv* = 1240/*λ*), where A was the absorption intensity, *R_∞_* was the reflection coefficient, and *λ* was the absorption wavelength [19]. The energy band gaps calculated from the intercept were 3.28 and 3.03 eV for TiO_2_ and TiO_2_@GO, respectively.

**Figure 2:**
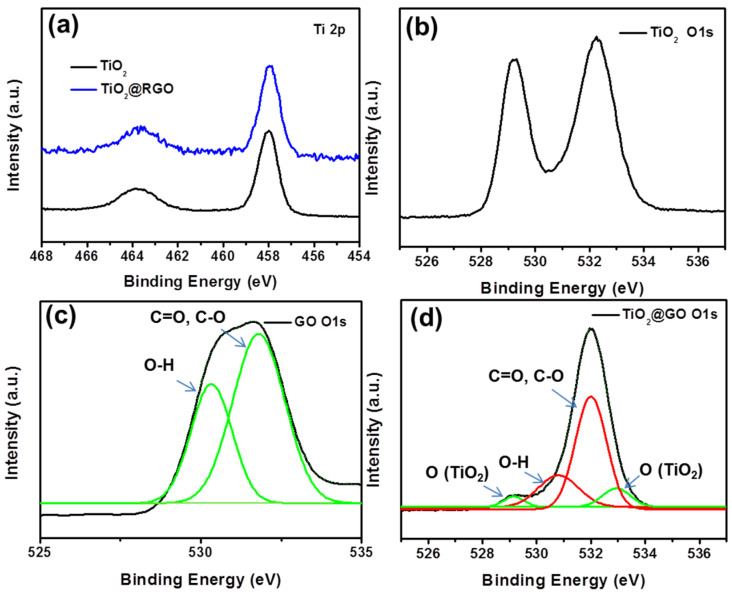
(**a**) XPS spectra of Ti 2p for TiO_2_ and TiO_2_@GO, XPS spectra of O 1s for (**b**) TiO_2_, (**c**) GO and (**d**) TiO_2_@GO.

The elemental compositions of TiO_2_, GO, and TiO_2_@GO was analyzed using XPS, a surface-sensitive spectroscopic technique, as shown in Figure 2. The TiO_2_ and TiO_2_@GO indicated two peaks at the binding energies of 458.5 and 464.3 eV for Ti 2p, which was assigned to the Ti 2p_1/2_ and Ti 2p_3/2_ [19]. The spectrum of TiO_2_ demonstrated two peaks of O 1s at 529.2 and 532.3 eV. The peaks of O 1s at about 530.3 and 531.7 eV were originating from the signal of O–H, C=O, and C–O of GO. The TiO_2_@GO exhibited four peaks at the binding energy of 529.2, 530.8, 532, and 533 eV, respectively, corresponding to the oxygen atoms of TiO_2_ and GO (oxygen-active functional groups) [12,17].

**Figure 3:**
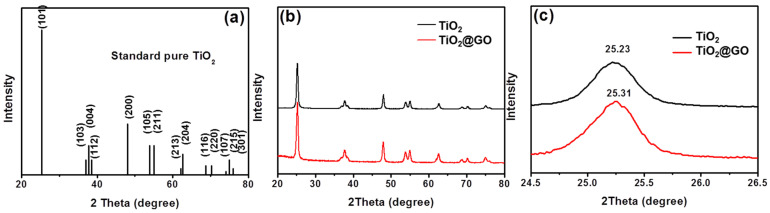
(**a**) X-ray diffractometer patterns of the standard pure TiO_2_ obtained from the MDI Jade. (**b**) X-ray diffractometer patterns of TiO_2_ and TiO_2_@GO, (**c**) (101) diffraction peaks of TiO_2_ and TiO_2_@GO.

XRD analysis [12,13,20] was carried out on as-milled TiO_2_ and TiO_2_@GO samples to identify the phase and crystalline structure of TiO_2_ and TiO_2_@GO. The results of the XRD were presented in Figure 3. Compared to the standard pure TiO_2_ obtained from the MDI Jade, the TiO_2_ and TiO_2_@GO showed a relative pure anatase phase [20]. The crystallinity of TiO_2_ and TiO_2_@GO were calculated by using the (101) diffraction peaks of anatase to study the influence of GO on the structure of obtained TiO_2_ [21]. The diffraction peak at 25.31° of TiO_2_@GO was higher than that at 25.23° of TiO_2_, revealing the existence of TiO_2_ at GO and a higher crystallinity of TiO_2_ for TiO_2_@GO. These changes were due to the introduction of a large number of oxygen-active functional groups of GO, which accelerated the crystallization and oxidation rate of TiO_2_ and led to promote the crystallinity of TiO_2_ at GO. Besides, the grain size of TiO_2_ was calculated by the MDI Jade (MDI, Livermore, CA, USA), which displayed the grain size of TiO_2_ and TiO_2_@GO with 34.6 nm and 28.4 nm, respectively. The increased crystallinity and smaller size of TiO_2_@GO were beneficial to enhance photocatalytic activity [12,13]. Furthermore, it was observed that the crystal planes distance (0.353 nm) of TiO_2_ in Figure 3 was a little larger than that (0.351 nm) of TiO_2_@GO, confirming that the GO matrix successfully induced the assembly of the in-situ growth TiO_2_. Combining the above crystallinity results, it could be further verified that the in-situ growth of TiO_2_ at GO almost optimized the crystal structure, increased the surface area, and fathered a little smaller crystal planes distance of TiO_2_ compared to pure TiO_2_ mainly due to the atomic space among a large number of oxygen-active functional groups of GO.

**Figure 4:**
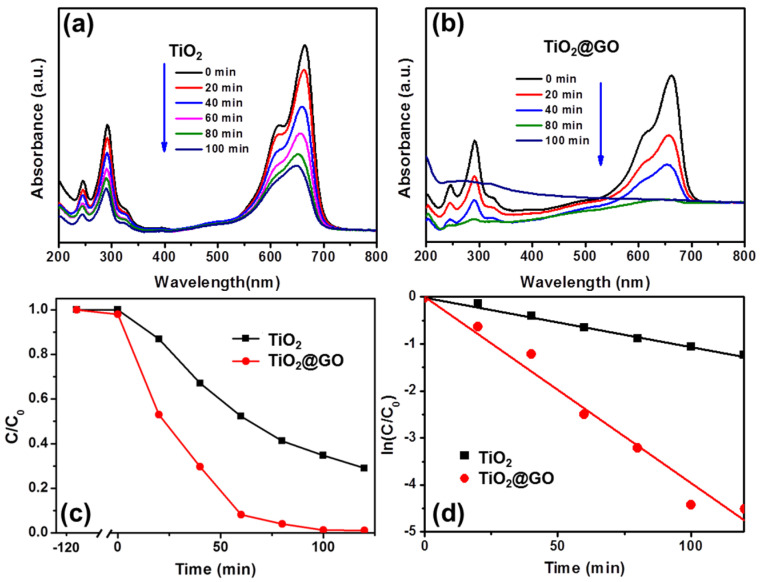
Methylene blue decomposition by (**a**) TiO_2_ and (**b**) TiO_2_@GO with the illumination of visible light; (**c**) Comparison of photocatalytic activities of TiO_2_ and TiO_2_@GO; (**d**) Dependence of ln(C/C_0_) on time.

The photocatalytic activity of TiO_2_ and TiO_2_@GO was studied by detecting the methylene blue decomposed in an aqueous solution under visible light. The methylene blue molecule was stable under the illumination of visible light without photocatalyst, because the concentration of methylene blue did not change after irradiation for 120 min in Figure 4. Before the photocatalysis experiments, the mixed solution with the methylene blue and photocatalyst was placed in a dark environment for 120 min to reach the adsorption equilibrium to preclude the adsorption of the photocatalyst. It seemed the adsorption ratios of TiO_2_ were very low because of the micrometer size surface. In contrast, TiO_2_@GO showed an obvious increase of photocatalyst degradation ratio of methylene blue might, due to the higher crystallinity morphology of TiO_2_ and the faster transmission of photogenerated electrons from TiO_2_ to GO. The presence of GO not only improved the adsorption capacity of target pollutants on the surface of the catalyst, but also promoted the transmission of photogenerated electrons from TiO_2_ to GO, causing the inhibition of the electron-hole recombination effectively.

**Figure 5:**
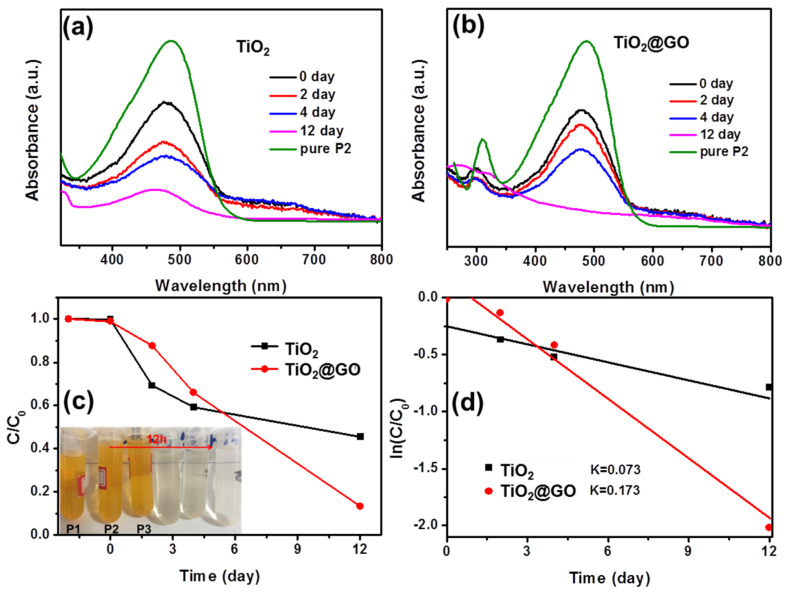
P2 decomposition by (**a**) TiO_2_ and (**b**) TiO_2_@GO with the illumination of natural light; (**c**) Comparison of photocatalytic activities of TiO_2_ and TiO_2_@GO; (**d**) Dependence of ln(C/C_0_) on time. The inset diagram showed water-soluble polymers degradation within 12 days under natural light.

**Figure 6:**
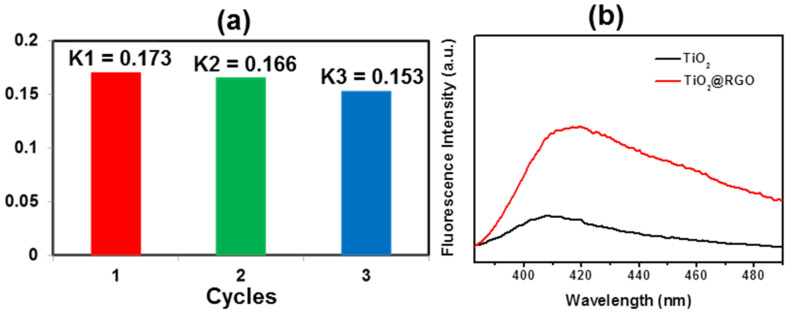
(**a**) The apparent first-order rate constant k versus times for TiO_2_@GO. (**b**) Fluorescence spectra of the visible light irradiated TiO_2_ and TiO_2_@GO.

Degradation of P2 aqueous solution was further proceeded to study the photocatalytic activity of TiO_2_ and TiO_2_@GO under natural light in Figure 5. The P2 was stable under the illumination of natural light because the concentration of P2 did not change after irradiation for two days without photocatalyst. Before photocatalysis experiments, to preclude the adsorption of the photocatalyst, the mixed solution with P2 and photocatalyst was also placed in a dark environment for 120 min to reach the adsorption equilibrium, and the remained concentration of polymer was regarded as the initial value. The absorbance intensity at about 480 nm of polymers decreased rapidly along with the illumination of natural light, and the color of solution almost disappeared after 12 days, confirming the effective photocatalytic decomposed activity of TiO_2_ and TiO_2_@GO. Moreover, it was obvious the TiO_2_@GO showed a higher photocatalyst activity than the TiO_2_, probably due to the higher crystallinity morphology and smaller size of TiO_2_ and the faster transmission of photogenerated electrons from TiO_2_ to GO. The presence of GO not only improved the adsorption capacity of target pollutants on the surface of the catalyst, but also promoted the transmission of photogenerated electrons from TiO_2_ to GO, which would reduce the recombination efficiency of photogenerated carriers. The photocatalytic degradation effect of TiO_2_@GO was also applied to P1 and P3, seen the Supplementary Materials, Figure S3. According to the Langmuir Hinshelwood model, the degradation process of P2 could be ascribed as a pseudo first-order kinetics reaction [22], and the corresponding apparent first-order rate constant (k) was calculated and revealed in Figure 6. The pseudo first-order kinetics was followed by the reactions, due to the linearity of all plots of degradation. The photocatalytic activity order was TiO_2_@GO > TiO_2_. The reason for the photocatalyst activity enhancement of TiO_2_@GO was that GO could act as acceptors of electrons, and effectively reduce the recombination center and enhance the separation efficiency of photocarries. Besides, in the recycle degradation of P2, the activity of TiO_2_@GO remained about 88% after three times recycle degradations in Figure 6, revealing its high photocatalytic stability.

**Scheme 3:**
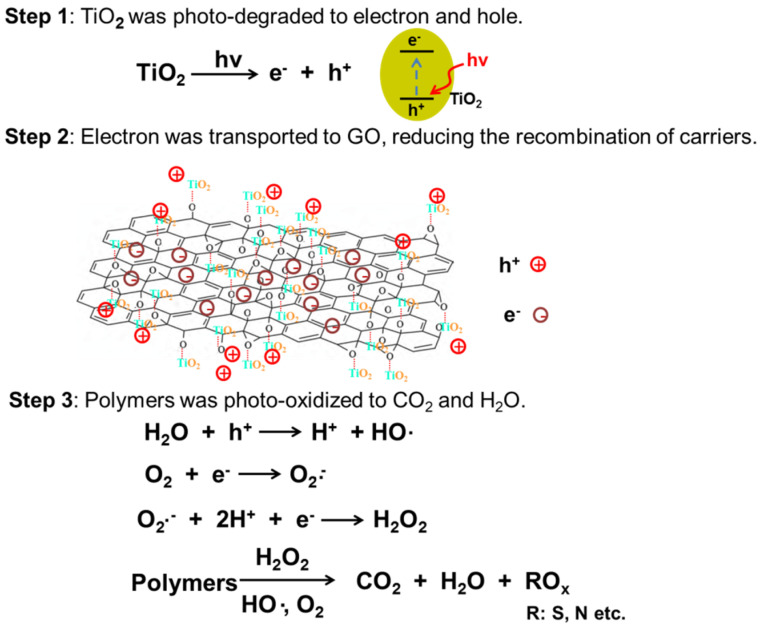
Schematic representations for the proposed reaction from polymers to CO_2_, H_2_O, and RO_X_ in natural environments.

In this study, it was strongly demonstrated the polymers underwent a photooxidative C–C bond cleavage process to get the CO_2_, H_2_O, and RO_X_ with TiO_2_@GO as the photocatalyst in a natural environment [23,24,25]. The photocatalytic properties of TiO_2_@GO were well-researched, as shown in Scheme 3 [26]. Upon natural light irradiation, charge separation on semiconductor TiO_2_ occurred. The generated electrons were effectively transported to the graphene sheets of GO, and the holes were accumulated within TiO_2_, resulting in decreasing the charge recombination. The accumulated holes were oxidative reaction with the H_2_O to produce •OH, and simultaneously the electron and H^+^ was also interacted with the O_2_ to bring the H_2_O_2_, leading to achieving the degradation of polymers.

To further study the effect of GO on the separation and recombination of photogenerated exciton from TiO_2_, the FL measurements were carried on, probing the production of •OH in the terephthalic acid aqueous of TiO_2_ and TiO_2_@GO [27,28,29]. The FL was excited by the reaction between the 2-hydroxyterephthalic acid and •OH radicals originating from the photogenerated exciton [30,31,32]. Upon natural light irradiation, charge separation on semiconductor TiO_2_ occurred. The generated electrons were effectively transported to the GO, and the holes were accumulated within TiO_2_, resulting in decreasing the charge recombination and obtaining more photoinduced separated electrons and holes. More photoinduced separated electrons and holes would produce more •OH radicals so that the separation and recombination of photogenerated exciton from TiO_2_ could be better studied through detecting the amount of •OH radicals. The TiO_2_@GO showed stronger FL intensity than TiO_2_ aqueous solution in Figure 6, indicating the more •OH radicals were produced in TiO_2_@GO, which confirmed GO could effectively decrease the generation of recombination centers, enhance the separation efficiency of the photoinduced electrons and holes, as well as increase the photocatalytic activity of TiO_2_@GO.

## 4. Conclusions

We had synthesized the in-situ growth of TiO_2_ at GO to form the TiO_2_@GO photocatalyst and studied its application in conjugated polymers’ degradation. The photodegradation process could be probed by UV-vis absorption originating from the conjugated backbone’s photosensitivity of polymers. The SEM and EDS element analysis was performed to prove that the TiO_2_ nanocrystals could be synthesized and self-assembled into a GO matrix by the in-situ growth method. The XRD analysis further confirmed that the in-situ growth of TiO_2_ at GO could optimize the crystal structure, increase the surface area, and father a smaller crystal planes distance of TiO_2_ mainly due to the atomic space among a large number of oxygen-active functional groups (hydroxyl, epoxy, carbonyl, carboxyl, etc.) of GO. Degradation of P2 aqueous solution was further studied by UV-vis absorption to detect the photocatalytic activity of TiO_2_ and TiO_2_@GO under the natural environment. The absorbance intensity at 480 nm of polymers reduced rapidly along with the illumination of natural light, and the color of solution almost disappeared after 12 days, confirming the effective photocatalytic decomposed activity of TiO_2_ and TiO_2_@GO. Furthermore, it was obvious the TiO_2_@GO showed a higher photocatalyst activity than TiO_2_, probably due to the higher crystallinity morphology and smaller size of TiO_2_ and the faster transmission of photogenerated electrons from TiO_2_ to GO. The FL analysis was further carried on to study the separation and recombination of photoinduced electrons and holes generating from TiO_2_. The TiO_2_@GO showed stronger FL intensity than TiO_2_, revealing the more •OH radicals were produced in TiO_2_@GO. It further confirmed GO could effectively decrease the generation of recombination centers, enhance the separation efficiency of photoinduced electrons and holes, as well as increase the photocatalytic activity of TiO_2_@GO.

## Data Availability

The data presented in this study are available on request from the corresponding author.

## Supplementary Materials

The following are available online at https://www.mdpi.com/article/10.3390/polym13132158/s1, Figure S1: GPC plots of P3, Figure S2: GPC plots of P2, Figure S3: P1’s degradation data, and the detailed information on the synthesis of all monomers.
