# Supplementary material for: In-Situ Synthesis of TiO2@GO Nanosheets for Polymers Degradation in a Natural Environment
Source: Polymers (Basel). 2021 Jun 30;13(13):2158. doi: 10.3390/polym13132158 (PMC8272197; doi:10.3390/polym13132158)
Supplement: Supplementary file 1 [file polymers-13-02158-s001.zip › polymers-1212716-supplementary.pdf]

Supplementary Materials

In-situ Synthesis of TiO_2@RGO Nanosheets for Polymers Degradation in a Natural Environment

Yueqin Shi[1*], Zhanyang Yu[1], Zhengjun Li[1], Yuan Sun[1], Xiaodong Zhao[1] , Yongjun Yuan[1] and Qi Zhang[1*]

1.Synthesis of all the monomers

1.1 Synthesis of 2-($C_4H_8SO_3Na$)-2*H*-benzo[1,2,3]triazole

Benzotriazole (28.6 mmol, 3.4 g) and tetrabutylammonium bromide (460 mg, 1.43 mmol) are dissolved in 140 mL anhydrous DMSO, which is degassed by N_2 for about 5 min. A 16 mL NaOH (14 g) aqueous solution is added via syringe. Then 1,4-butanesultone (3500 μL, 66.8 mmol) is added. After stirring for 3 hours at room temperature, the reaction mixture is poured into acetone. Through filtration and washed with acetone, the white precipitate is obtained. The product is purified by chromatography (reversed phase silica gel C-18, 10:1 H_2O:MeOH) to provide the white solid with 41% yield. ^{1}H NMR (500 MHz, D_2O) δ 7.53 (d, 2H), 7.86 (d, 2H), 4.79 (t, 2H), 2.89 (t, 2H), 2.24 (m, 2H), 1.74 (m, 2H). MS m/z calcd for $C_{10}H_{12}N_3SO_3Na$: 277 g/mol, found: 254 ($C_{10}H_{12}N_3SO_3^-$).

1.2. Synthesis of 4,7-dibromo-2-($C_4H_8SO_3Na$)-2*H*-benzo[1,2,3]triazole

BTN-$C_4H_8SO_3Na$ (0.73 mmol, 0.201 g) and an aqueous HBr solution (33 wt%, 1.3 mL) are added to the flask, and the mixture is stirred for one hour at 100 °C. Cooling the mixture to room temperature, bromine (0.33 g, 0.106 mL) is added. The solution is continued for 12 h at 80 °C. Cooling down to room temperature again, an aqueous solution of $NaHCO_3$ is added and the reaction is stirred with another 2 hours. The product is purified by chromatography (reversed phase silica gel C-18, 10:1 H_2O:MeOH) to provide the white solid 4 with 83% yield. ^{1}H NMR (400 MHz, D_2O) δ 7.59 (s, 2H), 4.88 (t, 2H), 2.90 (t, 2H), 2.25 (m, 2H), 1.74 (m, 2H). MS m/z calcd for $C_{10}H_{10}Br_2N_3SO_3Na$: 435 g/mol, found: 411.7 ($C_{10}H_{10}Br_2N_3SO_3^-$).

1.3. Synthesis of 1,2-diamino-3,6-dibromobenzene

$NaBH_4$ (20 mmol, 0.76 g) was added to the solution of 4,7-dibromo-benzo[1,2,5]thiadiazole (2 mmol, 0.588g) in 20 mL EtOH at 0 ºC. After stirring for 1 hour, the reaction was processed overnight. After removing the EtOH, the extraction was conducted with ether and water. The 1,2-diamino-3,6-dibromobenzene could be obtained after drying the organic phase with 80% yield. ^{1}H NMR (500 MHz, CD_3OD): δ= 6.5 (s, 2H)

1.4. Synthesis of 4,7-dibromo-1,2,3-benzotriazole

The aqueous solution of $NaNO_2$ (0.15 g, 1.65 mmol, 3 mL) is added to the solution of 1,2-diamino-3,6-dibromobenzene (0.4 g, 1.5 mmol) in CH_3COOH (6 mL). After stirring for 30 min at room temperature, precipitate is filtered and washed with water to afford 4,7-dibromo-1,2,3-benzotriazole (0.38 g) as a pink powder (yield is 85%). ^{1}H NMR (500 MHz, CD_3OD): δ= 7.56 (s, 2H)

1.5. Synthesis of 4,7-dibromo-2-(6-bromo-hexyl)-2*H*-benzotriazole

4,7-Dibromo-1,2,3-benzotriazole (1.45 mmol, 0.4 g) and *t*-BuOK (4.1 mmol, 0.46 g) were dissolved in 20 mL MeOH. At 60 ºC, 1,6-dibromohexane (14.5 mmol) was added to the solution. After stirring for 5 hours, the MeOH was removed and the residue was extracted with $CHCl_3$ and water. After concentration, purification by chromatography ($CHCl_3$:hexane = 1:1 vol) provided BTz-Br (0.37 g) in 59% yield. ^{1}H NMR (500 MHz, $CDCl_3$): δ = 7.43 (s, 2H), 4.81 (N-CH_2, 2H), 3.41 (Br-CH_2, 2H), 2.2 (-CH_2-, 2H), 1.84 (-CH_2-, 2H), 1.5 (-CH_2-, 2H), 1.42 (-CH_2-, 2H).

1.6. Synthesis of monomer with ammonium-end side chain

The monomers with bromo-end side chain are stirred in trimethylamine at 60 ºC for 2 days. Methanol is added to aid dissolution of ionic species. The volatiles are removed and the ionic-functionalized monomers can be dried over night at low pressure (99% yield). ^{1}H NMR (500 MHz, CD_3OD) for monomer with ammonium-end side chain 3: δ = 7.51 (s, 2H), 4.9 (N-CH_2-, 2H), 3.42 (N^+-CH_2-, 2H), 3.12 (-$N(CH_3)_3$, 9H), 2.24 (-CH_2-, 2H), 1.89 (-CH_2-, 2H), 1.51 (-CH_2-, 2H), 1.29 (-CH_2-, 2H).

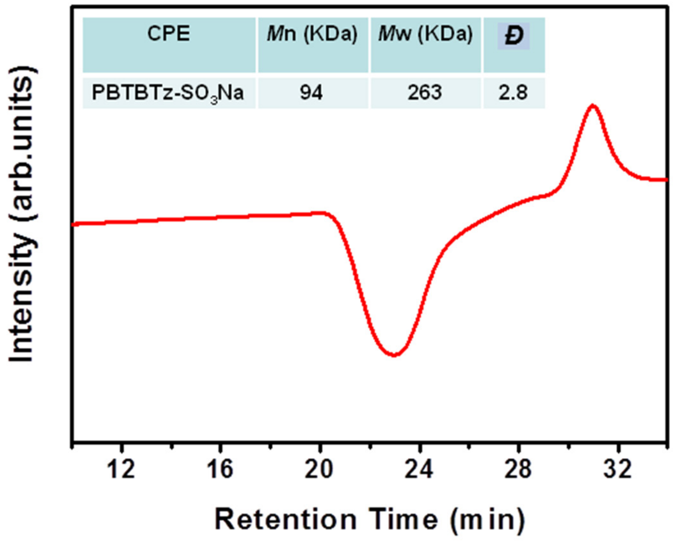

Figure S1. The GPC plots of the PBTBTz-SO_3Na (P3).

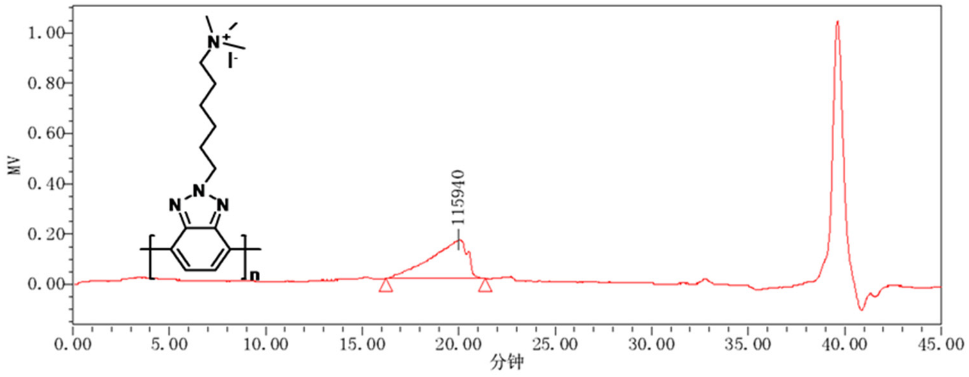

GPC 结果

	分布名	Mn (道尔顿)	Mw (道尔顿)	MP	Mz (道尔顿)	Mz+1 (道尔顿)	多分散性	MW 标记 1 (道尔顿)	MW 标记 2 (道尔顿)
1		157467	240626	115940	507419	1006844	1.528102		

Figure S2. The GPC plots of the PBTz-TMAI (P2).

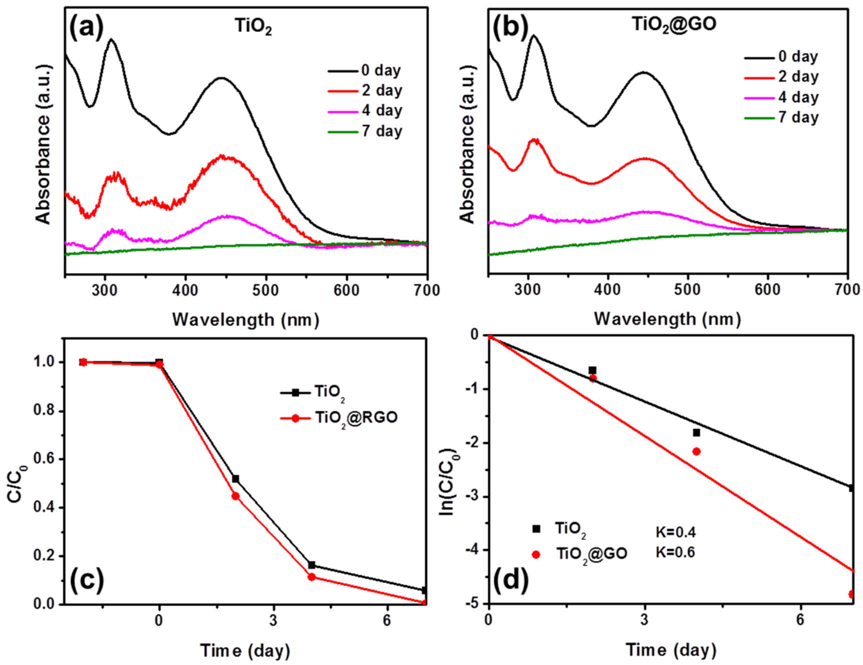

Figure S3. P1 decomposition by (a) TiO_2 and (b) TiO_2@GO with the illumination under nature light; (c) Comparison of photocatalytic activities of TiO_2 and TiO_2@GO; (d) Dependence of $\ln(C/C_0)$ on time.
